# Inflammatory Cytokine Profile and Plasticity of Brain and Spinal Microglia in Response to ATP and Glutamate

**DOI:** 10.3389/fncel.2021.634020

**Published:** 2021-04-06

**Authors:** Sam Joshva Baskar Jesudasan, Somnath J. Gupta, Matthew A. Churchward, Kathryn G. Todd, Ian R. Winship

**Affiliations:** ^1^Neurochemical Research Unit, Department of Psychiatry, Faculty of Medicine and Dentistry, University of Alberta, Edmonton, AB, Canada; ^2^Neuroscience and Mental Health Institute, University of Alberta, Edmonton, AB, Canada; ^3^Department of Biology and Environmental Sciences, Concordia University of Edmonton, Edmonton, AB, Canada

**Keywords:** microglia, adenosine tri phosphate, glutamate, inflammation, spinal cord, brain, conditioning

## Abstract

Microglia are the primary cells in the central nervous system that identify and respond to injury or damage. Such a perturbation in the nervous system induces the release of molecules including ATP and glutamate that act as damage-associated molecular patterns (DAMPs). DAMPs are detected by microglia, which then regulate the inflammatory response in a manner sensitive to their surrounding environment. The available data indicates that ATP and glutamate can induce the release of pro inflammatory factors TNF (tumor necrosis factor), IL-1β (interleukin 1 beta), and NO (nitric oxide) from microglia. However, non-physiological concentrations of ATP and glutamate were often used to derive these insights. Here, we have compared the response of spinal cord microglia (SM) relative to brain microglia (BM) using physiologically relevant concentrations of glutamate and ATP that mimic injured conditions in the central nervous system. The data show that ATP and glutamate are not significant modulators of the release of cytokines from either BM or SM. Consistent with previous studies, spinal microglia exhibited a general trend toward reduced release of inflammatory cytokines relative to brain-derived microglia. Moreover, we demonstrate that the responses of microglia to these DAMPs can be altered by modifying the biochemical milieu in their surrounding environment. Preconditioning brain derived microglia with media from spinal cord derived mixed glial cultures shifted their release of IL-1ß and IL-6 to a less inflammatory phenotype consistent with spinal microglia.

## Introduction

Microglia have the capacity to respond to pathogens, insults, and injuries that disrupt the homeostasis of the CNS (Helmut et al., 2011). They are able to respond to a wide variety of environmental stimuli due to their repertoire of receptors that can sense damage-associated molecular patterns (DAMPs) and pathogen-associated molecular patterns (PAMPs) in the central nervous system (CNS) (Kigerl et al., 2014; Gadani et al., 2016). Adenosine triphosphate (ATP) and glutamate are examples of DAMPs that are released into the extra cellular milieu in response to various injuries or perturbations of CNS, including cell death due to stroke, spinal cord injury, traumatic brain injury (Kigerl et al., 2014; Gadani et al., 2016). ATP and glutamate are well known for their role as chemotactic agents that recruit microglia to the site of injury (Duan et al., 2008; Liu et al., 2009). ATP has also been shown to induce upregulation of pro inflammatory factors such as TNF (tumor necrosis factor), IL-1β (interleukin 1 beta), and NO (nitric oxide) by microglia through purinergic receptor pathways (Helmut et al., 2011; Rodrigues et al., 2015). Similarly, glutamate has been shown to induce pro-inflammatory factors such as TNF, IL-1β, and NO in microglia through glutamate receptors (Pocock and Kettenmann, 2007; Helmut et al., 2011; Murugan et al., 2013). However, most studies that investigated these DAMPs used specific receptor agonists or antagonists or used non-physiological concentrations (≥10 fold above the physiological concentrations) to determine their effects on microglia in culture (Vincent and Maiese, 2000; Dai et al., 2010). ATP and glutamate can activate ionotropic [ATP: P2X1-7, glutamate: AMPA(Glu2/3), kainate (Glu5)] and metabotropic receptors (ATP: P2Y1-6,11-14, glutamate: mGlu1-8) on microglia (Färber and Kettenmann, 2006; Liu et al., 2009; Helmut et al., 2011; Murugan et al., 2013). Previously, it was shown that 1 mM glutamate induces release of TNF through AMPA (GluR 2-4) and kainate (GluR5) receptors (Noda et al., 2000). The Group II mGlurR2 and 3 specific agonist DCG-IV also induces TNF release by microglia (Taylor et al., 2005). Interestingly, selective inhibition of group II mGlu5 reduces TNF release by LPS-activated microglia (Byrnes et al., 2009). In the present study, physiologically relevant concentrations of ATP and glutamate that mimic injury were used to test if they have a differential effect on brain-derived (BM) and spinal cord-derived (SCM) primary microglia. Previous studies in models of rat ischemia and TBI have shown that glutamate concentrations of up to 10 μM occur in the uninjured CNS extracellular milieu, and that this concentration is increased to ≥30 μM after ischemic injury or TBI (Marini and Paul, 1992; Ueda et al., 1993; Dai et al., 2010; Hinzman et al., 2010). Extracellular glutamate concentrations of more than 100 μM induce neurotoxicity (Marini and Paul, 1992). While there is no general consensus on ATP concentration in the extracellular milieu during homeostasis, it has been suggested that after an injury a 500 μM concentration and above occurs (Burnstock, 2006; Seeland et al., 2015) and several studies have utilized a 1 mM concentration of ATP to measure the effect of ATP on microglia *in vitro* (Sibley and Lefkowitz, 1985; Verderio and Matteoli, 2001; Kawamura et al., 2004; Lai, 2010; Harada et al., 2011). Microglial phenotypes are dependent on region of origin (Lai et al., 2012) age, sex and environment (Sorge et al., 2011; Lai et al., 2013; Grabert et al., 2016). Notably, in spared nerve injury (SNI) model, spinal cord microglia (SCM) and brain microglia posses different regional features which affects their response to injury with upregulation of spinal BDNF as compared cortex (Xuan et al., 2019). SCM have a reduced inflammatory profile in response to activation by lipopolysaccharide (LPS) relative to microglia derived from the brain (BM) (Baskar Jesudasan et al., 2014). However, the responses of SCM (relative to BM) to physiological stimulation with ATP and glutamate have not been investigated. Given that previous data suggest a less inflammatory phenotype in SCM, this study tested the hypotheses that physiological activators such as ATP and glutamate would induce a reduced inflammatory profile in SCM compared to BM. Previously, we have shown that microglia from different regions of brain (hippocampus and thalamus) that are exposed to conditioned media from striatum acquired an inflammatory profile similar to that of microglia originally derived from the striatum (Lai, 2010). This suggests that microglia are a highly plastic population of cells. Hence, we also hypothesized that regional heterogeneity is not fixed and that BM exposed to SCM condition media would move toward an inflammatory profile similar to that of SCM.

To test the first hypothesis, BM and SCM from postnatal Sprague-Dawley rat pups were activated *in vitro* with ATP (1 mM) *in vitro* (Lai, 2010) and glutamate. Glutamate concentrations of different concentrations were selected to match *in vivo* conditions including physiological concentrations in the uninjured brain parenchyma (10 μM), or pathophysiological concentrations that model rat brain parenchyma after ischemic or TBI injury (30 μM) and excitotoxic injury (Choi and Rothman, 1990; Marini and Paul, 1992; Ueda et al., 1993; Dai et al., 2010; Hinzman et al., 2010). To test the second hypothesis, BM were incubated in conditioned media from brain and spinal cord mixed glia (BMix CM and SMix CM, respectively) to replicate the extracellular environment in which BM and SCM were cultured. BM conditioned in BMix cm and SMix cm were activated with ATP, glutamate (10, 30, 100 μM) and LPS (1 μg/ml) and pro-inflammatory factors released were measured.

## Methods

### Media and Reagents

Hanks Balanced Saline solutions (HBSS), Dulbecco's Modified Eagle Medium—Hams'F12 nutrient mixture (DMEM-F12), DMEM-F12 with HEPES (DMEM-F12/HEPES), 0.25% trypsin-EDTA, fetal bovine serum (FBS), and Penicillin-Streptomycin (P/S) were from Gibco (ThermoFisher Scientific, Burlington, ON). ATP, glutamate, lidocaine HCl, Triton X-100, LPS, and sodium nitrite standard solution were from Sigma (Oakville, ON).

### Primary Mixed Glia Preparation

All animal protocols were conducted in accordance with Canadian Council on Animal Care Guidelines and approved by the Animal Care and Use Committee: Health Sciences for the University of Alberta. Brains and spinal cords for establishing primary mixed glial cultures were obtained from postnatal day one or two male Sprague-Dawley (SD) rat pups as previously described (Lai and Todd, 2008; Churchward and Todd, 2014). The SD rat pups were euthanized, their brains (four) and spinal cords (twenty) were dissected and placed in dissection buffer (HBSS with 200 U/mL penicillin, 200 μg/mL streptomycin). Meninges and blood vessels were removed under a dissection microscope. Tissues were cut into small pieces and incubated in 0.25% Trypsin-EDTA for 25 min at 37°C, and collected by centrifugation (2,000 × g, 2 min). Trypsin was inactivated with maintenance media (DMEM/F12 supplemented with 10% FBS and 200 U/mL penicillin, 200 μg/mL streptomycin) and tissues were dissociated by trituration in maintenance media and centrifuged at 2,000 × g for 2 min. The brain and spinal cord cell pellets were re-suspended in maintenance media and seeded at equal density into cell culture-treated T75 flasks coated with poly-L-lysine. Cells were maintained in a 37°C, 5% CO_2_ humidified incubator with maintenance media replaced twice weekly.

### Microglia Isolation by Lidocaine HCl Isolation Method

Microglia were isolated from primary mixed glial cultures at 21 days *in vitro* by modification of the lidocaine HCl shaking method (Siao and Tsirka, 2002; Lai and Todd, 2008). At 24-h prior to isolation cultures were refreshed with DMEM/F-12 supplemented with 10% FBS, and immediately before isolation this medium was collected, filtered (0.22 μm), and diluted 1:1 with DMEM/F-12 to make conditioned medium. Brain and spinal mixed glial cultures at 21 days *in vitro* were refreshed with DMEM-F12/HEPES with 10% FBS at 37°C 30 min before lidocaine HCl treatment. Lidocaine HCl was added to the primary mixed glial culture to a final concentration of 15 mM in the media, the cultures were incubated for 3 min and shaken for 7 min in an orbital shaker at 50 rpm at 37°C. Microglia were collected from the media by centrifugation (2,000 × g, 2 min at room temperature). Cell pellets were gently re-suspended in 1 mL of conditioned media using 1 mL pipette tip. Cell suspensions were washed by adding 9 mL of their respective conditioned media and centrifuged at 2,000 × g for 2 min at room temperature. The cell pellets were again re-suspended by trituration in their respective conditioned media and the cells were counted and seed at a density of 1 × 10^5^ cell/mL in 48 or 24 well poly-L-lysine coated polystyrene plates. The microglia were allowed to settle in the plates for 10 min at 37°C in a 5% CO_2_ incubator after which non-adherent cells were washed gently with DMEM at 37°C and respective conditioned media were added to the BM and SCM and the mixture were allowed to recover overnight. Conditioned media were replaced with fresh DMEM/F-12 immediately prior to treatments.

### Nitric Oxide (NO)

NO release was measured indirectly by quantifying the stable metabolite nitrite in culture media using a method described by Griess (1879). Media were collected 24 h after treatment, 100 μL of treatment or control media were added to a 96-well plate in duplicates followed by 50 μL each of 1% sulphanilamide (in 3N HCl) and 0.02% N-napthylethylenediamine per well. Absorbance was read at 540 nm and the amount of nitrite metabolite was interpolated from a set of standards measured in parallel.

### Enzyme Linked Immunosorbent Assays (ELISA)

Commercial ELISA kits were used to measure TNF, IL-1β, and IL-6 in media (DuoSet, R&D Systems Minneapolis, USA). ELISA procedures were carried out according to manufacturer protocols.

### Statistics

Statistical analyses were carried out using two-way ANOVA followed by Sidak's methods to test for significance between treatment groups. n represents a single independent experiment (i.e., an independent culture preparation) with a minimum of three technical replicates. Each technical replicate represents a well in a 12, 24, or 48 well culture plate. All statistical analyses were done using Graphpad Prism version 8.3.0.

## Results

### Secretion of Pro-inflammatory Effectors by BM and SCM Exposed to ATP

**Figure 1:**
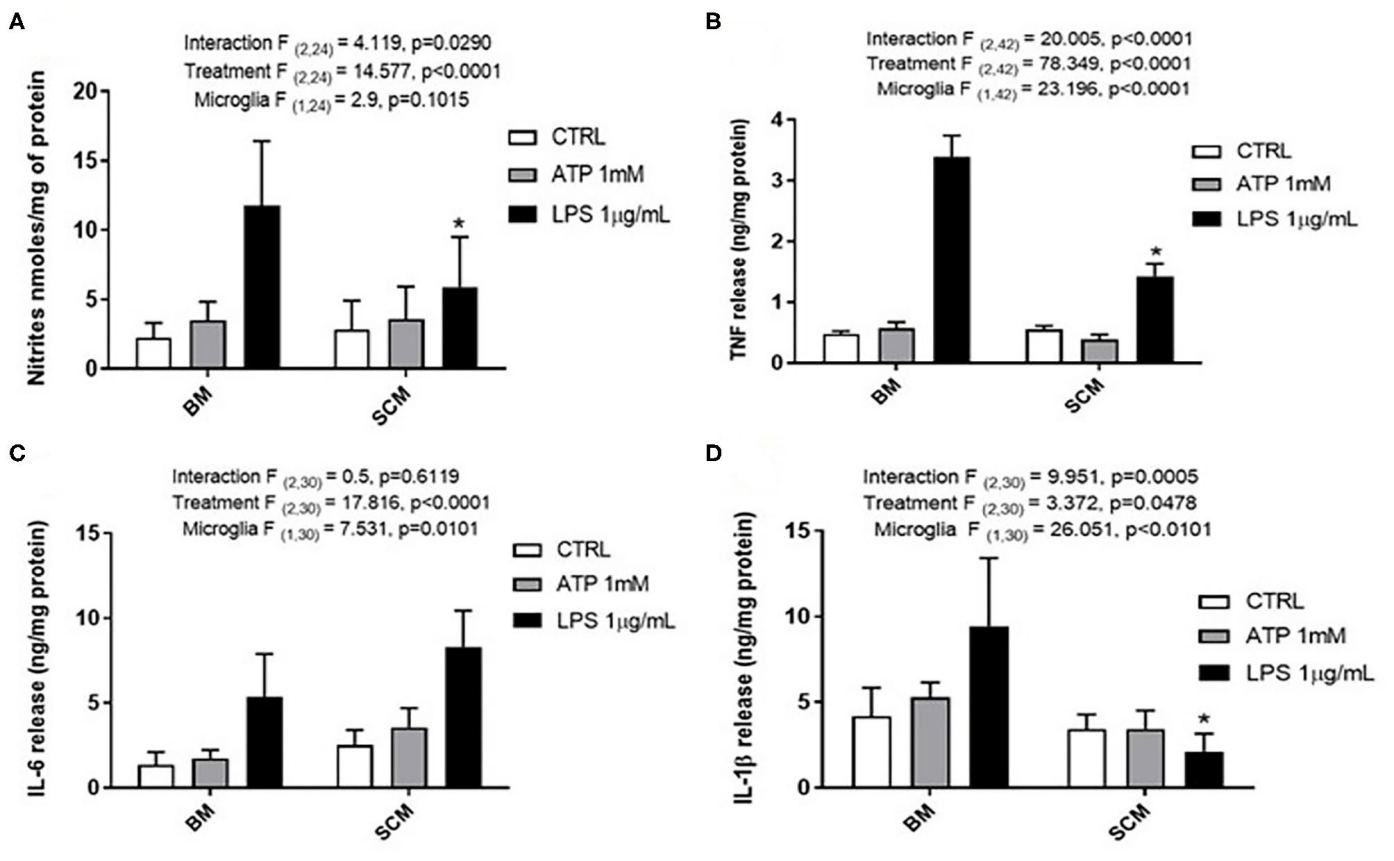
Release of the pro-inflammatory effectors by ATP activated BM and SCM. **(A)** Two-away ANOVA identified significant interaction between microglia and treatment for NO and a main effect of treatment for NO. However, Sidak *post-hoc* test revealed a significant difference between BM LPS and SCM LPS, * represents *p* < 0.05, *n* = 5, where *n* represents the number of independent experiments (an independent experiment is a separate microglia preparation). **(B,C)** Two-away ANOVA identified significant interaction between microglia and treatment TNF and IL 6. TNF released had a significant difference between BM LPS and SCM LPS was revealed by the Sidak *post-hoc* test, * represents *p* < 0.05, *n* = 8 for TNF and *n* = 6 for IL6. **(D)** Two-away ANOVA identified significant interaction between microglia and treatment for IL-1β and a significant main effect of microglia. A Sidak *post-hoc* test revealed a significant difference between BM LPS and SCM after LPS treatment. * represents *p* < 0.05 for comparison, *n* = 6. ATP treatment did not induce a significant difference in the release of inflammatory factor NO, TNF, IL 6, IL−1 β between BM and SCM ATP treatments. Bars represent mean ± s.e.m.

ATP is commonly released in the extracellular milieu after injury to the CNS (Helmut et al., 2011; Rodrigues et al., 2015; Gadani et al., 2016). To investigate phenotypic differences between SCM and BM in response to this endogenous activator, isolated SCM and BM microglia were treated with ATP. Previous studies suggest that ATP concentrations of 1 mM are sufficient to induced a pro-inflammatory profile (NO- Nitic oxide, TNF- Tumor necrosis factor, IL-1β–Interleukin 1β and IL-6—Interleukin 6) in BM (Sibley and Lefkowitz, 1985; Verderio and Matteoli, 2001; Kawamura et al., 2004; Lai, 2010; Harada et al., 2011). BM and SCM were therefore treated with 1 mM ATP, and pro-inflammatory factors (NO, TNF, IL-6, IL-1β) released into cell culture media were measured using the Greiss assay (NO) and ELISAs (TNF, IL-6, IL-1β) (Figure 1, Table 1). Two-away ANOVA significant interaction between microglia and treatment for NO, TNF, IL-1β, a significant main effect of treatment for release of NO, TNF, IL-1β and a significant main effect of microglial origin was observed for TNF, IL-6 and IL-1β, the data is summarized in Table 1. Sidak *post-hoc* test revealed that NO, TNF and IL-1β released by LPS (1 μg/ml) activated SCM was significantly less than that of BM (BM LPS vs. SCM LPS: NO *p* = 0.042, TNF *p* < 0.001, IL-1β *p* < 0.0001). No other significant comparisons were found, suggesting that group differences were largely driven by differential responses to LPS activation rather than ATP treatment.

**Table 1:** Two-way ANOVA details for effect of ATP on SCM and BM.

**Pro-inflammatory effectors**	**ATP**					
	**Interaction**		**Treatment**		**Microglia**	
	**F (Dfn, Dfd)**	***p***	**F (Dfn, Dfd)**	***p***	**F (Dfn, Dfd)**	***p***
TNF	(2, 42) = 20.005	*p* < 0.0001	(2, 42) = 78.349	*p* < 0.0001	(1, 42) = 23.196	*p* < 0.0001
IL-6	(2, 30) = 0.5	*p =* 0.6119	(2, 30) = 17.816	*p* < 0.0001	(1, 30) = 7.531	*p =* 0.0101
IL-1β	(2, 30) = 9.951	*p =* 0.0005	(2, 30) = 3.372	*p =* 0.0478	(1, 30) = 26.051	*p* < 0.0001
NO	(2, 24) = 4.119	*p =* 0.0290	(2, 24) = 14.577	*p* < 0.0001	(1, 24) = 2.900	*p =* 0.1015

### Secretion of Pro-inflammatory Effectors by BM and SCM Exposed to Glutamate

**Figure 2:**
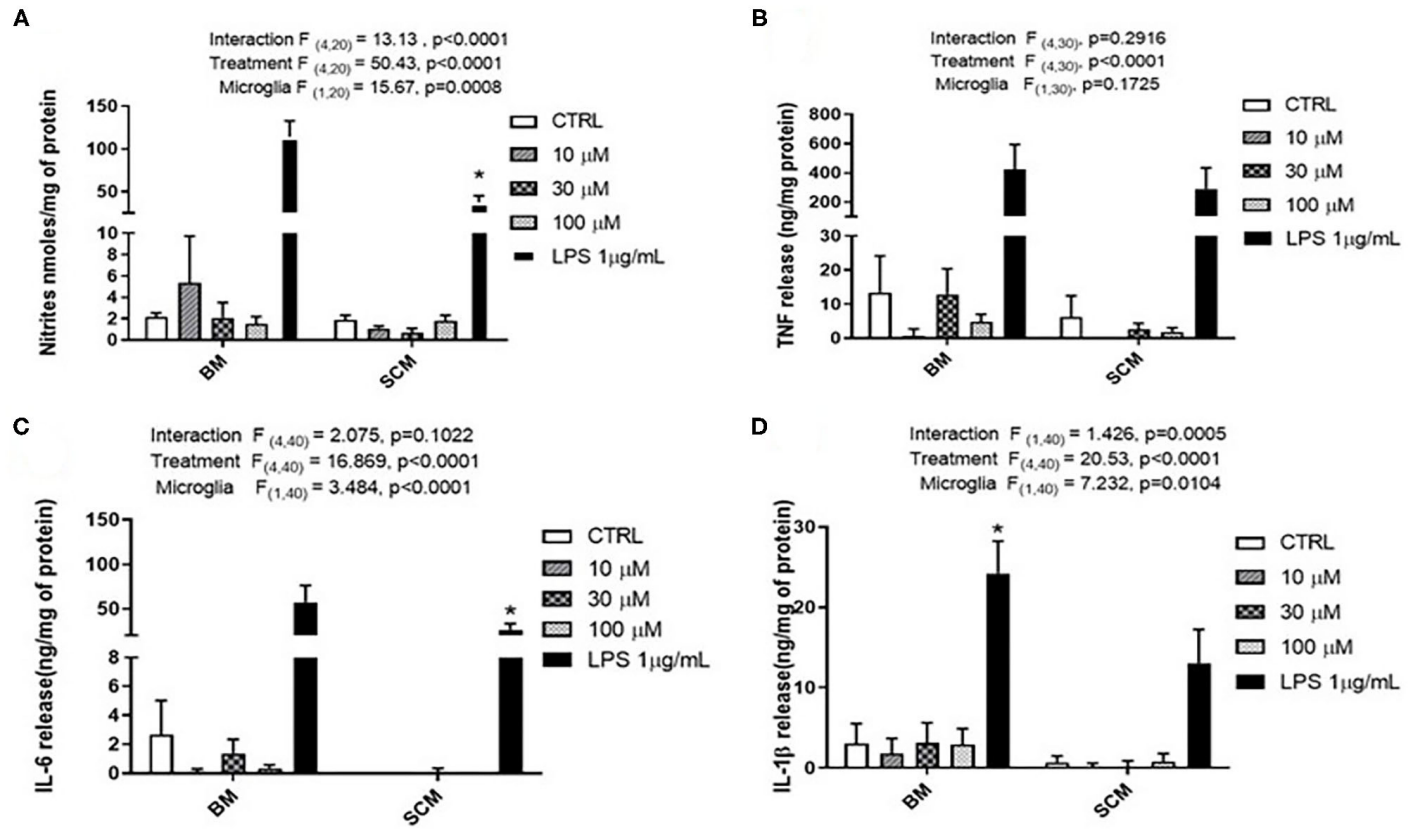
Release of the pro-inflammatory effector by BM and SCM exposed to glutamate. **(A)** Two-way ANOVA identified significant interaction between microglia and treatment for NO. There was also a significant main effect of microglia and treatment, *n* = 3 (where n represent the number of independent experiments i.e., separate microglia preparations). **(B,C)** Two-way ANOVA revealed a significant main effect for glutamate treatment for TNF. *n* = 4 for TNF and *n* = 5 for IL-6. **(D)** Two-way ANOVA revealed a significant main effect for microglia and treatment (*n* = 5). * represents *p* < 0.05 a significant difference between respective LPS treatments revealed by the Sidak *post-hoc* test. Bars represent mean ± s.e.m.

Glutamate agonists have been shown to induce pro or anti-inflammatory profile in a receptor dependent manner in microglia (Vincent and Maiese, 2000; Dai et al., 2010). However, direct study of microglia phenotype in response to physiological concentrations of glutamate has not been frequently investigated. Here, 10 μM (representing physiological levels of glutamate in CNS extra cellular milieu), 30 μM (levels present in *in vivo* ischemic injury), 100 μM (levels present at excitotoxicity injury sites) (Choi and Rothman, 1990; Ueda et al., 1993) were selected as concentrations for treatment of BM and SCM (Figure 2). Two-way ANOVA identified significant interaction between microglia and treatment for NO, IL-6, IL-1β, and TNF. A significant main effect of treatment for TNF, IL-6, and IL-1β and significant main effects of microglia for IL-1β, IL-6 and NO were also detected (Table 2). Sidak *post-hoc* analysis revealed that NO released by SCM LPS was significantly less than that of LPS treated BM (BM LPS vs. SCM LPS *p* < 0.0001, Figure 2A), and that LPS mediated IL-6 and IL-1β release by SCM was significantly less than that of BM (BM LPS vs. SCM LPS *p* < 0.05) (Figures 2C,D). While a qualitative trend toward reduced release of all inflammatory mediators in response to glutamate in SCM was suggested, *post-hoc* comparisons did not identify statistically significant differences in cytokine release between BM and SCM in response to different glutamate concentrations.

**Table 2:** Two-way ANOVA details for effect of glutamate on SCM and BM.

**Pro-inflammatory effectors**	**Glutamate**					
	**Interaction**		**Treatment**		**Microglia**	
	**F (Dfn, Dfd)**	***p***	**F (Dfn, Dfd)**	***p***	**F (Dfn, Dfd)**	***p***
TNF	(4, 30) = 1.302	*p =* 0.2916	(4, 30) = 38.127	*p* < 0.0001	(1, 30) = 1.954	*p =* 0.1725
IL-6	(4, 40) = 2.075	*p =* 0.1022	(4, 40) = 16.869	*p* < 0.0001	(1, 40) = 3.484	*p* < 0.0001
IL-1β	(1, 40) = 1.426	*p =* 0.0005	(4, 40) = 20.53	*p* < 0.0001	(1, 40) = 7.232	*p =* 0.0104
NO	(4, 20) = 13.13	*p* < 0.0001	(4, 20) = 50.43	*p* < 0.0001	(1, 20) =15.67	*p =* 0.0008

### Secretion of Pro-inflammatory Effectors by BM Exposed to BMix and SMix CM

**Figure 3:**
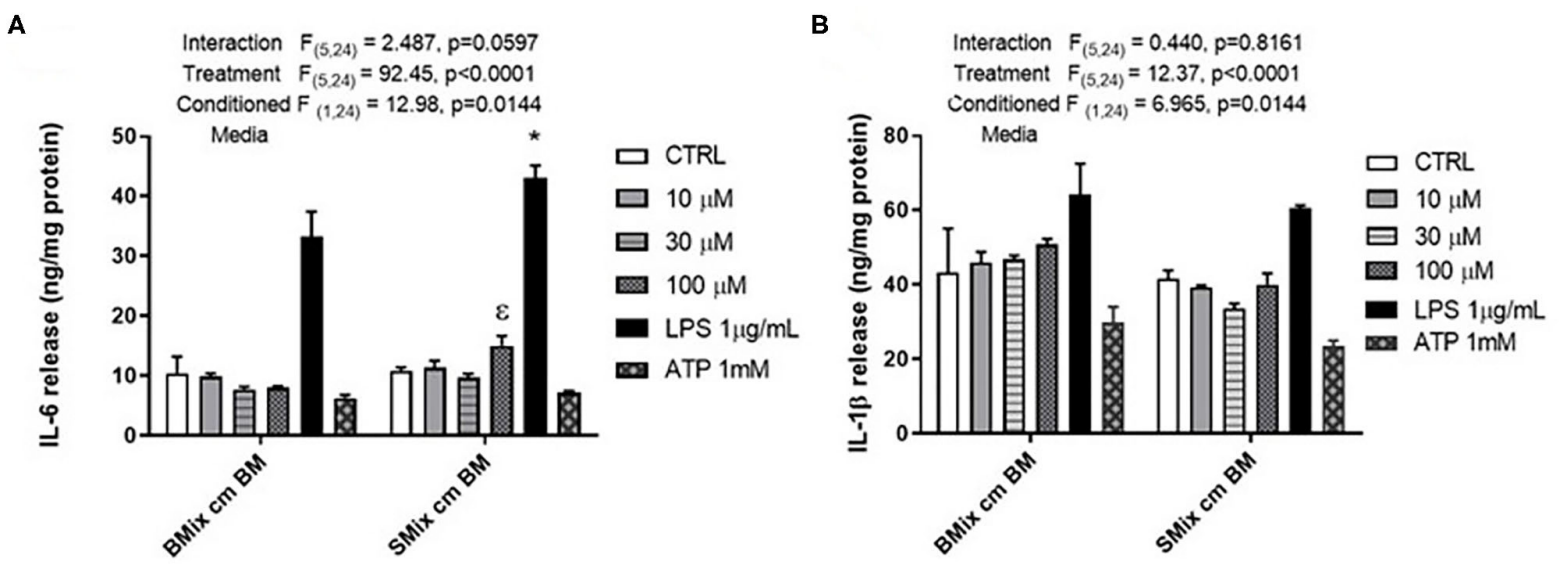
Secretion of the pro-inflammatory cytokines by BM exposed to BMix CM and SMix CM. **(A)** Two-way ANOVA also revealed a significant main effect of conditioned media and treatment for IL-6 release. ε represents significant difference for 100 μM glutamate treatment (*p* = 0.0480) between brain mixed glia conditioned media (BMix CM) and spinal mixed glia conditioned media (SMix CM) * represents *p* < 0.05 significant difference between LPS treatment for BMix CM and SMix CM as revealed by the Sidak *post-hoc* test, *n* = 3 where n represent the number of independent experiments. **(B)** Two-way ANOVA revealed a significant main effect of conditioned media and treatment for the release of IL 1β. However, the Sidak *post-hoc* test did not reveal any significant differences between BMix CM and SMix CM (*n* = 3). Bars represent mean ± s.e.m.

Microglia are brain resident macrophage which has hematopoietic stem cell origin. Microglia from different maturation state when transplanted into microglia deficient mice, acquires microglia gene expression (Bennett et al., 2018). Moreover, ramified morphology of microglia is enhanced by culturing on astrocytic monolayer or astrocyte conditioned media (ACM) (Bohlen et al., 2017; Zhang et al., 2020). BM are highly plastic cells capable of adapting to immediate environment and their phenotype is dictated not only by genetics but also their immediate environment. Therefore, the pro-inflammatory profile of SCM may be due to the conditioning by spinal cord mixed glia culture media. We hypothesized that conditioning BM with SMix CM (spinal cord mixed glia conditioned media) would alter the concentrations of inflammatory factors released by BM. To test the hypothesis, BM were incubated in SMix CM or BMix CM (brain mixed glia conditioned media) prior to treatment with ATP, glutamate, or LPS. BM incubated in BMix CM and SMix CM were activated with glutamate (10, 30, 100 μM), ATP (1 mM), LPS (1 μg/ml) and the release of IL-6 and IL-1β was measured. Two-way ANOVA indicated that conditioned media and treatment had a significant main effect for IL-6 and IL-1β release. Overall, the data suggest that BM release more IL-6 and less IL-1β when incubated with SMix media relative to BMix media. Sidak *post-hoc* tests suggest that IL-6 released in response to LPS was significantly greater in BM incubated in SMix CM (BMix CM BM LPS vs. SMix CM BM LPS, *p* < 0.0032) (Figure 3A, Table 3). Moreover, BM release of IL-6 in response to 100 μM glutamate was significantly greater when incubated in SMix CM (BMix CM BM 100 μM vs. SMix CM BM 100 μM, *p* = 0.0480) (Figure 3A) and suggested an overall reduction in IL-1β release with SMix CM (Figure 3B). Sidak *post-hoc* comparisons did not reveal any significant difference between individual BMix CM and SMix CM treatment groups for IL-1β (Figure 3B).

**Table 3:** Conditioned media-mediated cytokine profile of BM and SCM.

**Pro-inflammatory effectors**	**Condition media**					
	**Interaction**		**Treatment**		**Condition Media**	
	**F (Dfn, Dfd)**	***p***	**F (Dfn, Dfd)**	***p***	**F (Dfn, Dfd)**	***p***
IL-6	(5, 24) = 2.487	*p =* 0.0597	(5, 24) = 92.45	*p* < 0.0001	(1, 24) = 12.98	*p =* 0.0014
IL-1β	(5, 24) = 0.440	*p =* 0.8161	(5, 24) = 12.37	*p* < 0.0001	(1, 24) = 6.965	*p =* 0.0144

## Discussion

ATP and glutamate are known DAMPs that potentiate the microglial inflammatory response. In Spinal cord injury (SCI) and Traumatic Brain Injury (BI), these DAMPs can induce a differential inflammatory profile that may vary as a function of their concentration in the parenchyma. This study focused whether these mediators at physiological concentrations induce the release of inflammatory cytokines by BM and SCM. Our results suggest that ATP and glutamate do not induce significant release of pro-inflammatory factors such as NO, TNF, IL6, and IL-1β in either SCM or BM. However, our data do not preclude a role for these activators in the inflammatory response to brain injury.

### ATP Mediated Cytokine Profile of BM and SCM

Previous studies suggest that SCM have a reduced inflammatory phenotype in response to LPS exposure relative to BM. In this study, we found no differences in the release of the proinflammatory factors NO, TNF, IL-6, or IL-1β between BM and SCM treated with ATP (1 mM) (Figures 1A–D). This data suggests regional heterogeneity (i.e., microglia from brain vs. microglia from spinal cord) does not influence ATP (purinergic receptors) mediated activation of microglia, unlike LPS where the SCM inflammatory profile was reduced compared to that of BM (a result replicated here). This data supports the inference that ATP by itself may have a primary role as a chemoattractant rather than an immunomodulator (Honda et al., 2001; Wu et al., 2007). Moreover, previous studies have also shown that combination of DAMPs (ATP + LPS) induces a much more rapid induction of inflammatory cytokine IL-1β than just LPS (peaked at 5 h post treatment vs. at 25 h) (Sanz and Virgilio, 2000). The data from this study indicates that origin of microglia (brain or spinal cord) does not alter their inflammatory behavior in response to ATP alone. ATP may instead act in combination with other DAMPs to evoke induction of inflammatory cytokine release after an injury, in addition to a role as a chemoattractant.

### Glutamate Mediated Cytokine Profile of BM and SCM

Previous studies shown that 1 mM glutamate induced release of TNF through AMPA (GluR2-4) and kainate (GluR5) receptors (Taylor et al., 2005). Similarly, Group II m GlurR2 and GlurR3 specific agonist DCG-IV induce TNF release by microglia (Taylor et al., 2005). However, 1 mM glutamate does not mimic the concentration of glutamate at the site of an ischemic injury or TBI (Marini and Paul, 1992; Ueda et al., 1993; Dai et al., 2010; Hinzman et al., 2010). The purpose of this glutamate study was to test if glutamate can induce the release of inflammatory factors at physiological concentrations mimicking ischemic and excitotoxicity injury, and to test if glutamate could induce differential release of inflammatory factors by BM and SCM. Glutamate at physiological concentrations did not induce significant release of pro-inflammatory factors (TNF and IL-6) and there was no significant difference between BM and SCM release in individual treatment groups (Figures 2B,C). However, an overall reduction in NO and IL-1β release by SCM was observed in glutamate experiments (Figures 2A,D). Interestingly, it has been shown that glutamate can induce chemotaxis in microglia (Liu et al., 2009). Thus, glutamate at physiological concentrations may also play a primary role as a chemoattractant in addition to its role as mediator of inflammation. At higher concentration of glutamate, the BM and SCM did not show any significant change in release of proinflammatory factors (TNF, IL-6, and NO). One possible explanation could be related to the ability of microglia to uptake glutamate and convert it to glutathione, thus protecting from oxidative stress (Persson et al., 2006). This effect may be linked to TNF- α release, as it has been shown to potentiate the expression of GLT-1 and leading to increased glutamate uptake (Persson et al., 2005). Glutamate treatment did not show any morphological change in microglia, suggesting that glutamate is not directly involved in initiating inflammatory response from microglia (Goshi et al., 2020).

### Conditioned Media-Mediated Cytokine Profile of BM and SCM

All microglia in the CNS originate from the yolk sac during embryogenesis. This suggests that all microglia in CNS are genetically very similar (Ginhoux and Prinz, 2015). However, microglia are very versatile and studies have shown that they can have different phenotypes (surface receptor expression, pro-, and anti- inflammatory factors release) in distinct regions of brain (Lai, 2010). For example, the P2XR7 and P2YR12 expression is higher in striatum than in other regions of the brain (Lai, 2010). This suggests that microglia adapt to their immediate environment, potentially due to paracrine signaling from neighboring cells that alters the extracellular milieu.

The conditioned media experiments were designed to answer the question of whether BM exposed to SMix CM would adopt a more SCM-like phenotype, when compared to SCM from earlier experiments and compared to BM exposed to BMix CM. Notably, SMix CM significantly increased release of IL-6 compared to BMix CM (Figure 3A), suggesting that the environmental milieu can significantly affect phenotype for certain inflammatory molecules. This observed increase in release of IL-6 is consistent with a shift to a more SCM-like phenotype by BM, as evidenced by enhanced IL-6 release in SCM in Figure 1 (though elevated IL-6 from in response to LPS was not observed in Figure 2), and in previously published data on LPS induced release of IL-6 from SCM compared to BM (Baskar Jesudasan et al., 2014). Similarly, an overall main effect of conditioned media suggesting reduced IL-1β release was observed after incubation of BM with SMix CM. While the LPS group comparison did not reach significance, the data are generally consistent with observations of SCM treated with LPS (in Figures 1, 2, and in Baskar Jesudasan et al., 2014) and suggests BM may adopt a more SCM like phenotype in SMix CM. The conditioned media experiments therefore provide further evidence that BM phenotype is plastic and can be modified by varying the extracellular environment, thus supporting the hypothesis that not only the region of origin but also the immediate environment determine the phenotype of microglia. This further supports the postulate that microglial phenotype is not fixed by region of origin and that immediate environment plays a crucial role in the diversity of microglial phenotypes after perturbation or injury to CNS. The data also aligns well with previous studies that demonstrated that the inflammatory profile of microglia is dependent on the severity of injury, where microglia were beneficial to outcome of mild injury (Lai and Todd, 2008; Lai et al., 2013). However, the phenotype of BM in SMix CM reflects an intermediate phenotype between isolated SCM and BM, supporting that both intracellular and extracellular factors regulate cytokine release.

This study investigated whether ATP and glutamate induce a differential inflammatory profile in SCM vs. BM and addressed whether these mediators at physiological concentrations induce the release of inflammatory cytokines by BM and SCM. Our study is one of the few studies to examine the effects of physiological and pathological concentrations of glutamate on microglia. Our results suggest that ATP and glutamate do not induce significant release of pro-inflammatory factors such as NO, TNF, IL-6, and IL-1β. However, our data do not preclude a role for these activators in the inflammatory response to brain injury. Both ATP and glutamate are involved in chemotaxis of microglia and can potentially recruit microglia to the site of injury or perturbation (Wu et al., 2007; Harada et al., 2011). Moreover, previous findings demonstrated that ATP in combination with LPS induced faster maturation and release of intracellularly accumulated IL-1β (Sanz and Virgilio, 2000), suggesting a modulatory role that was not tested here. Earlier studies showed that exposing microglia from thalamus and hippocampus to conditioned media from striatum induces a striatum-like phenotype in the microglia (Lai, 2010). Similarly, we found that SMix CM altered the response profiles of BM compared to BMix CM, including increased release of IL-6 and reduced release of IL-1β in response to LPS activation and increased release of IL-6 following glutamate treatment (Figures 3A,B). The data from this study suggests that SMix CM altered the inflammatory response from BM, with BM adopting a phenotype more consistent with SCM. We did not probe whether SCM exposed to BM would adopt a more BM-like phenotype. However, we would postulate that the effects of the extracellular milieu on cytokine release from microglia reflect inherent microglial plasticity not dependent of region of origin. As such, we would predict that this plasticity would be bidirectional and SCM conditioned with BMix CM would adopt a more BM-like phenotype. Overall, our data suggest that ATP and glutamate at physiological concentration do not induce an inflammatory cytokine release in BM and SCM and provide further support that the phenotype of BM as well as SCM *in vitro* is determined by both region of origin and immediate environment in addition to other factors such as age and sex (Sorge et al., 2011; Lai et al., 2013; Grabert et al., 2016). Age of animals from which cultures are derived also modulate microglial activity, as phenotype in culture varies as a function of age (Hart et al., 2012). Of note, microglia isolated form 3 days old mice have different patterns of protein expression compared to microglia isolated from 21 days old mice (Crain et al., 2013). Available literature on *in vitro* age dependent expression of purinergic receptors indicates that the mRNA expression of P2XR and P2YR changes with age (Crain et al., 2009). Thus, the response properties observed in BM and SCM isolated from day old pups here may differ in cultures from older animals.

## Data Availability Statement

The raw data supporting the conclusions of this article will be made available by the authors, without undue reservation.

## Ethics Statement

The animal study was reviewed and approved by Animal Care and Use Committee—Health Sciences at the University of Alberta.

## Author Contributions

SJ, IW, MC, and KT conceived and designed experiments. SJ collected data. SJ, SG, and MC performed data analysis and figure preparation. All authors contributed to manuscript preparation.

## Conflict of Interest

The authors declare that the research was conducted in the absence of any commercial or financial relationships that could be construed as a potential conflict of interest.
